# Supplementary material for: A mathematical model to quantify the effects of platelet count, shear rate, and injury size on the initiation of blood coagulation under venous flow conditions
Source: PLoS One. 2020 Jul 29;15(7):e0235392. doi: 10.1371/journal.pone.0235392 (PMC7390270; doi:10.1371/journal.pone.0235392)
Supplement: Attachment — Submitted filename: Response to reviewers.pdf [file pone.0235392.s001.pdf]

A mathematical model to quantify the effects of platelet count, shear rate, and injury size on the initiation of blood coagulation under venous flow conditions

A. Bouchnita, K. Terekhov, P. Nony, Y. Vassilevski, V. Volpert

Authors' responses for the questions of the reviewers

We are grateful to the editor and the reviewers for their valuable suggestions. We have taken them into consideration to prepare the revised version. Below, we provide point-by-point responses to the questions raised by the reviewers. A color-marked version of the manuscript with the amended text is attached after list of answers.

Reviewer #1:

The manuscript proposes a model describing the formation of the thrombus in venous vessel. The model is extended from the previous work of the authors, by adding the participation of the platelets. To identify the parameters, the authors adopt another so-called thrombin generation model which does not include the advection of the flow. This gives the value of the parameters by fitting the output data to the experimental data. Using the fitted parameters, their model can reproduce the threshold response to the shear rate in clotting initiation. Further more, they deviate a simplified 1D equation from their model, which could also capture the threshold feature. The simplified model may provide a cheap way to estimate the influence of the various parameters on the thrombus formation.

We thank the Reviewer for the evaluation of our manuscript and the comment on our work. We have tried to address all the concerns raised by the reviewer in the revised version of the manuscript.

Major comments:

1) The control parameter, shear rate, is implied by a constant pressure in this work. However, the flow will change with the growth of the thrombus in the presence of constant pressure boundary. Thus the shear rate is not a well controlled parameter in the simulations. While the compared experiment used the constant velocity or flux rate as the imposed flow.

The goal of the study is to determine the shear rate value that prevents the initiation of the clot rate. Shear rate can depend on the pressure difference, but it can also depend on the flow rate as it is the

case in the experiments. In both cases, the shear rate changes only if clot growth is initiated. The initial shear rate could be controlled by either changing the pressure difference or the flow rate depending on the chosen boundary conditions. However, we have used a constant pressure condition instead of a constant flow rate condition to test the adequacy of the model to the physiologically relevant conditions. Indeed, the constant pressure condition is required to observe the scenario of complete venous occlusion. We have added the following sentences suggesting that varying the pressure drop was used to control the initial value of the shear rate (page 11 of the revised manuscript):

"We vary the value of the pressure drop to control the initial shear rate. Then, the value of the shear rate can also change if clot growth is initiated. However, in this case we intend to determine the value of the shear rate that prevents the initiation of the coagulation process. Therefore, we determine the value of the initial shear rate that prevents blood clotting by running consecutive simulations."

2) The system is symmetrical. But the asymmetrical colour map appears in Fig. 4 and Fig. 5. Could the authors explain the reason?

Slight axial asymmetry of the numerical solution shown in Fig. 4 and Fig. 5 is caused by grid effects: the numerical model is based on the 3D grid shown in Fig.3 which is not axially symmetric, and the isosurfaces produced by the visualizer Paraview in Figs. 4,5 may be also affected by the grid asymmetry:

3) In Fig. 6 a, the value of platelet count is not given for the platelets rich plasma case. I guess that it is $150 \times 10^9 L^-1$. This gives the threshold shear rate at about $80 s^-1$. While the experiment in ref 10 used the value of platelet count as $300 \times 10^9 L^-1$ and gave the threshold shear rate at about $80 s^-1$. Speculated from Fig. 7, the threshold shear rate for $300 \times 10^9 L^-1$ platelet count would be around $160 s^-1$ in the simulations. Could the authors explain this deviation between the experimental observation and the simulation.

We thank the reviewer for this important remark. In the initial version of the manuscript, we did not pay attention that the experiments in Ref. 10 consider the platelet count equal to $300 \times 10^9 L^-1$. As a result, we refitted the parameter k_1 which is the kinetic constant of prothrombin activation by active platelets. The value that yields the best resuts is $k_1 = 7 \times 10^{-5} L s^{-1}$ which is approximately half of the previously considered value. To prepare the revised manuscript, we have repeated all numerical simulation and updated Figures 6, 7, and 8. With these changes, the threshold value for PRP is around 80 s^{-1} as observed in the experiments. We have added the following sentece to indicate the considered platelet density for PRP:

"We consider that for PRP, platelet density is equal to 300 $\times$ 10^9 L^{-1} which is the value considered in the experiments [11]."

4) As the authors indicate that the length of the tube will significantly influence the clotting initiation time, could the author do more discussion about the effect of the tube length and the tube diameter as well?

The shear rate of a pressure driven Newtonian fluid in a pipe is given by:

$$\gamma = \frac{\Delta p D}{4 \nu L},$$

where L is the length of the vessel and D is its diameter. As a result, if the pressure drop is kept constant, then increasing the diameter of vessel or decreasing the length of the tube reduce the shear rate. This will lead to an increase in the clotting initiation time. We have added the following sentences to describe the effect of shear rate and clot growth initiation (page 15 of the revision):

"We speculate that for a constant pressure drop, decreasing the length of the vessel or increasing its diameter result in an increase of the shear rate that prevents clotting initiation."

Minor comments:

1) The quality of the figures should be improved. For example, the colour bar can not been seen, the legend in Fig. 3a miss $\times$, the label of sub-figures sometimes is capital while sometimes not..

Following the suggestions of the reviewer, we have improved the quality of figures in the revision.

2) Some typo was found. Such as 'thrombin [II]' below the equation 18. I think this should be 'prothrombin [II]'. Please do a careful checking, since the model contains a lot of parameters, small typo may make the reader lost.

Following the suggestion of the reviewer, We have made a careful proofreading and corrected the typos.

Reviewer #2:

This work presents a quantitative study of the effects of platelet count, wall shear rate, and injury size on the initiation of blood coagulation based on the analysis of the model and on numerical simulations. First, they described the mathematical modelling of venous thrombus growth under flow and derived a simplified model of thrombin distribution. Then, through numerical simulations, they studied the relationship between platelet count and blood coagulability, the shear rate threshold dependence on the density of platelets, and the effect of TF surface area on the initiation of blood clotting. The results in this work should be of interest to PLOS ONE community. However, I think this paper still has some issues to be addressed before it can be accepted.

We thank the reviewer for the evaluation of our work.

1): As I know, the spatiotemporal model of thrombus development and thrombin generation model have been previously proposed. What's the difference between them and models presented in this work?

We agree that there are different spatio-temporal models of blood coagulation in the literature. Our model was developed in such a way that it can be reduced to a thrombin generation model, and then to a 1-D thrombin distribution model which yields approximately the same results. As a result, our model can be used to estimate the coagulability of blood, to simulate clot growth under the flow, and to derive analytical relationship between the factors affecting clot initiation. We have added this sentence to the discussion to emphasize on this feature of the model (page 16 in the revision):

"Indeed, the main feature of the presented model in comparison with the rest of spatio-temporal models is the possibility to derive from it a thrombin generation model and a 1-D thrombin distribu-

tion model. As a result, it was possible to simulate the development of the thrombus under flow, to estimate the coagulability of blood, and to derive analytical estimates for the relationships between the initiation of coagulation and the various model parameters. Furthermore, there exist very few 3D continuous models that describe hemodynamics, the coagulation biochemistry, and platelet kinetics at the same time."

2): Authors simplified the thrombin distribution with 1D model, however, this is a 3D problem. If it is appropriate to assume the distribution of thrombin in x-z plane (using the coordinate in the work)?

To address this question, we first need to ensure that the 3D model can be simplified to a 2D model because it is symmetrical with respect to the azimuth angle when we consider the cylindrical coordinates. Then we derive the 1D model from this 2-D model by considering the evolution of the maximum height reached by the clot. It corresponds to the propagation of the thrombin wave and characterizes the rate of vessel occlusion. It is an approximation and comparison with the 3D model justifies its applicability. We have added the following sentence to the manuscript explaining this point (page 9 of the revised manuscript):

"Hence, the model approximates the evolution of the maximal height reached by the distribution of thrombin over time. Therefore, it provides a tool to describe the rate of vessel occlusion."

Minor:

1): In the introduction, author should clarify why the platelet play different roles in arterial and venous flow.

We have added the following sentences to the introduction summarizing the differences between the roles of platelets in arterial and venous flows:

"In arteries, platelets initiate thrombus formation by adhering to the subendothelium and recruiting other platelets. As a result, we refer to the thrombi developed in arteries by 'white clots' because they consist mainly of platelets. In venous flows, fibrin propagates during the early stages and prevents the platelets from aggregating. The formed thrombi are called 'red clot' because they are formed mainly by the fibrin mesh and RBCs [2]. In this case, platelets accelerate the production of the fibrin mesh by secreting procoagulant factors upon their activation."

2): In the fig 3. The results have good agreement with the experiments. However, the simulation exlcudes the effects of RBCs in the venous flow. In the Discussion, author states that RBCs will accelerate the margination of platelets, if it is possilbe to include the RBCs effect in the model.

The experiments exclude RBCs as well because they are performed on plasma and not the whole blood, which excludes blood cells except platelets in the case of PRP. Therefore, it is not needed to introduce them in the model for this work. We had already indicated this in the original version of the manuscript:

"Note that red blood cells (RBCs) and their effects are not considered in the present work because the microfluidic experiments that are used to validate the model are performed on blood plasma which does not contain these cells."

Technically, it is possible to introduce RBCs to the model. Although there exist very few models of blood coagulation that consider erythrocytes. We have added this sentence that summarizes the techniques by which RBCs can be introduced:

"The marginalization of platelets by RBCs can be introduced implicitly by using discrete methods such as dissipative particle dynamics [43] and immersed boundary methods [44]. Furthemore, blood can be simulated as a multi-constituent fluid with one phase for the plasma with RBCs and the other phase representing the plasma with platelets [45]."

3): The occlusion of vessel is presented in fig 6. How to estimate the occlusion, please clarify this.

In the model, we consider that the thrombus is formed when the concentration of thrombin exceeds 200 nM. We observe that thrombin production amplifies as soon as its concentration reaches that value. We have already mentioned how we estimate vessel occlusion in the original version of the manuscript:

"Therefore, we consider that the percent of the vessel that is occluded corresponds to the regions where thrombin exceeds a certain threshold $T^* = 200\ nM$."

We have added the following sentence to the description of Fig. 6 to make its understanding easier for the reader:

"Occluded regions are domains where the concentration of thrombin exceeds 200 nM"

4): Author just use two cases (different platelet count) in microfluidc capillary. This is not enough to make comparison.

We understand that the relatively low number of available microfluidic experiments is one of the main limitations of the current study. In Fig. 8, we have added a comparison with data for the threshold response of the clotting initiation to the size of the TF patch. In the future, we plan to validate the model by comparing its results with other experimental studies. We have highlighted this purpose by adding this sentence:

"Furthermore, we will also use the model to reproduce other microfluidic experiments corresponding to other blood clotting scenarios."

A mathematical model to quantify the effects of platelet count, shear rate, and injury size on the initiation of blood coagulation under venous flow conditions

Anass Bouchnita[1 ‡*], Kirill Terekhov [2‡], Patrice Nony [3], Yuri Vassilevski [2, 4, 5], Vitaly Volpert [2, 6, 7, 8],

1 Ecole Centrale Casablanca, Casablanca, Morocco
2 Marchuk Institute of Numerical Mathematics, Russian Academy of Sciences, Moscow, Russia
3 Services de Pharmacologie Clinique, Hospices Civils de Lyon, Lyon, France
4 Sechenov University, Moscow, Russia
5 Moscow Institute of Physics and Technology, Russia
6 Institut Camille Jordan, Université Lyon 1, Villeurbanne, France
7 INRIA team Dracula, INRIA Lyon La Doua, Villeurbanne, France
8 Peoples' Friendship University of Russia (RUDN University), Moscow, Russia

‡These authors contributed equally to this work.
* anass.bouchnita@centrale-casablanca.ma

Abstract

Platelets upregulate the generation of thrombin and reinforce the fibrin clot which increases the incidence risk of venous thromboembolism (VTE). However, the role of platelets in the pathogenesis of venous cardiovascular diseases remains hard to quantify. An experimentally validated model of thrombin generation dynamics is formulated. The model predicts that a high platelet count increases the peak value of generated thrombin as well as the endogenous thrombin potential (ETP) as reported in experimental data. To investigate the effects of platelets density, shear rate, and wound size on the initiation of blood coagulation, we calibrate a previously developed model of venous thrombus formation and implement it in 3D using a novel cell-centered finite-volume solver. We conduct numerical simulations to reproduce *in vitro* experiments of blood coagulation in microfluidic capillaries. Then, we derive a reduced one-equation model of thrombin distribution from the previous model under simplifying hypotheses and we use it to determine the conditions of clotting initiation on the platelet count, the shear rate, and the plasma composition. The initiation of clotting also exhibits a threshold response to the size of the wounded region in good agreement with the reported experimental findings.

1 Introduction

Blood coagulation can be initiated when the tissue factor (TF) is exposed to the bloodstream [1]. In arteries, platelets initiate thrombus formation by adhering to the subendothelium and recruiting other platelets. As a result, we refer to the thrombi developed in arteries by 'white clots' because they consist mainly of platelets. In venous flows, fibrin propagates during the early stages and prevents the platelets from aggregating. The formed thrombi are called 'red clots' because they are formed mainly

by the fibrin mesh and RBCs [2]. In this case, platelets accelerate the pro duction of the fibrin mesh by expressing negatively charged phospholipids up on their activation. Studies, quantifying the relationship between platelets and the risk of venous thrombosis, have not attracted a lot of attention, because platelets play a more important role in arterial thrombus formation. Still, an elevated platelet count is considered to be a risk factor for the incidence of venous thromboembolism (VTE). In fact, it was shown that a platelet count higher than $350 \times 10^9\ L^{-1}$ can be considered as an independent risk factor for VTE [3] and that it is also associated with the hypercoagulability of blood [4]. These clinical studies were confirmed by *in vitro* assays such as thrombin generation which measure the coagulability of blood samples from individual patients. In these assays, it was observed that both the peak thrombin and the endogenous thrombin potential (ETP) values increase as the density of platelets in plasma grows [5]. During thrombus formation, platelets are recruited into the thrombus and further increase its hydraulic resistance [6]. Hence, the role of platelets in the formation of venous thrombus consists in increasing the coagulability of blood and reducing the porosity of the thrombus. Quantitative studies clarifying the relationships between platelets, blood flow, as well as the other elements of hemostasis, are of paramount importance to develop better treatment strategies for VTE.

Blood coagulation is a complex process that leads to thrombus formation inside blood vessels. It can be initiated when the endothelial tissue is damaged and tissue factor (TF) is exposed to bloodstream. In this early phase of hemostasis, platelets adhere to exposed collagen and express TF as well as other clotting factors upon their activation [7]. TF form a complex with FVII and FVIIa that activates the blood clotting factors FIX and FX near the damaged tissues [8]. Factor FXa convert prothrombin (FII) into thrombin (FIIa) in the plasma which results in the generation of thrombin, the most important factor in the coagulation cascade. Thrombin converts fibrinogen into fibrin which subsequently forms the fibrin clot [9]. In the early stages of thrombus formation, blood clotting is only initiated when a sufficient amount of generated thrombin is able to trigger the amplification phase of the coagulation cascade. During this phase, thrombin activates the clotting factors FV, FVIII, and FXI. The latter activates FIX which converts FX into its active form. At the same time, FVIIIa forms a complex with FIXa that activates FX. Then, FXa and FVa forms a complex called prothrombinase which activates prothrombin. This results in a self-amplifying cycle of thrombin generation. This cycle can only be stopped by few mechanisms such as the inhibition of thrombin production by antithrombin and activated protein C (APC) as well as its mechanical removal by blood flow. Aggregated platelets reduce the velocity of blood flow and produce thrombin as well as other clotting factors which accelerate the coagulation process.

Various types of studies were conducted in order to understand the underlying mechanisms that lead to the initiation of thrombus growth under the flow. Theoretical investigations begin already during the 19th century with the Virchow's triad that divides the factors leading to thrombosis into three categories: blood stasis, hypercoagulability, and alterations affecting the vessel wall [10]. Experimental studies not only validated these hypotheses but also explored the relationship between the different factors involved in venous thrombosis pathogenesis. In an important work that falls into this category [11], *in vitro* experiments were conducted using microfluidic capillaries. A patch containing TF was inserted inside a tube with rigid walls where blood was flowing due to the action of a syringe pump. Initiation of coagulation time was measured for normal pooled plasma (NPP) and platelet-rich plasma (PRP) under different values of shear rate and for different sizes of the TF patch. The work is of particular importance because it provides quantitative results that show the existence of a threshold response for the initiation of the clotting process under different conditions.

There have been several methods that were used in the mathematical modelling of the kinetics of blood coagulation and the dynamics of thrombus growth [12]. The complexiy of each of the developed models depend on the extend of the scope it intends to achieve [13]. In order to evaluate the coagulability of blood samples from individual patients, researchers typically use deterministic models consisting of ordinary differential equations (ODEs) describing the concentrations of clotting factors over time [14–17]. The densities of platelet subtypes (activated and inactivated) can also be introduced as ODEs in these models. The advantage of this approach is the ability to compare the modelling results (thrombin generation curves) with the output of major coagulation assays such as the thrombin generation test. It is also possible to analyse these models mathematically to understand the dynamics of blood coagulation. Furthermore, these works can be used as a basis to develop more complex models that describe the spatial dynamics of thrombus growth by adding advection and diffusion terms to models in order to capture the spatial distribution of clotting factors. The kinematics of blood flow can be captured using continuous models such as the Navier-Stokes equations [18] or discrete methods such as Dissipative Particle Dynamics [19,20]. The discrete approach is more appropriate to simulate the dynamics of platelet aggregation as observed in arterial cardiovascular events [2]. In order to describe the interaction between thrombus growth and hemodynamics, some studies consider clot as a permeable medium whose hydraulic resistance depends on the concentration of fibrin polymer [21], or the density of platelets [22]. Another approach consists in the modelling of the effects of the clot on the viscoelastic properties of blood flow [23]. In general, the models describing the formation of venous thrombi focus on the distribution of clotting factors in plasma while those studying the development of arterial white thrombi are primarily interested by the dynamics of platelets activation and aggregation [24]. Although mathematical modelling provides an important tool for the qualitative understanding of the underlying mechanisms behind thrombus growth, it is difficult to properly use it to quantify the effects of these mechanisms.

Among the challenges that face the use of mathematical modelling as a tool to conduct robust quantitative studies of thrombus are the disparities in the values of blood coagulation kinetic constants reported in the literature. These disparities stem their origin from different settings in which these parameters are measured, which calls into question the validity of the predictions made by the models of thrombus formation [25]. Furthermore, difficulties in performing *in vivo* and *in vitro* experiments of thrombus growth represent an important challenge, that must be overcome for the validation of computational models. Moreover, most of the spatial models of thrombus formation are too complex and computationally expensive which limits their usefulness in conducting quantitative studies.

This work is devoted to data-driven modelling of thrombus formation under venous flow. Mathematical models of blood coagulation are formulated and used in order to investigate the effects of platelets on blood coagulability and on the initiation of thrombus formation under various conditions. We sketch numerical basics of the models since their computational aspects are beyond the scope of the present paper. The adopted approach consists in reproducing the available experimental data by calibrating the developed models and conducting numerical simulations. The calibrated models are simplified and analyzed to predict the clotting response under different conditions. This method is of particular importance because it can be used to perform quantitative studies for a wide range of model parameters without running long consecutive simulations using more sophisticated models. To evaluate the effect of platelet count on blood coagulability, we propose a new model, describing the kinetics of thrombin generation. The kinetic constants of the model were fitted to reproduce the observed thrombin generation in assay kits. We measure the values of the thrombin peak and the

endogenous thrombin potential (ETP) for different densities of platelets and confirm the existing relationship between high platelet count and hypercoagulability. The next part of the paper is devoted to the study of the effects of platelets on thrombus growth under venous flow. To do this, we calibrate a previously developed model of clot growth under flow [26,27] to realistically simulate the *in vitro* experiments [11] in 3D settings. Among the main features of the considered model is the possibility to reduce it to a one-equation model yielding approximately the same solution. This offers the opportunity to conduct quantitative studies and easily evaluate the conditions of the initiation of clotting on various parameters. In particular, we estimate the thresholds of the shear rate that prevent clotting for different platelet count values as well as the critical size of the injured area leading to the initiation of clot growth.

2 Mathematical modelling of venous thrombus growth under flow

2.1 Spatiotemporal modelling of thrombus development

2.1.1 Advection-diffusion-reaction equations for blood factors and platelet densities

We extend the previously developed model of clot growth dynamics [26,27] to include the aggregation of platelets. The model consists of advection-diffusion-reaction equations describing the spatiotemporal distributions of blood coagulation factors and platelet subtypes during thrombus development. A similar model was previously used to describe venous thrombus formation in rheumatoid and psoriatic arthritis [28] as well as during anti-vitamin K therapy [29]. The part of the coagulation cascade considered in the model is shown in Figure 1. First, we describe the concentration of prothrombin:

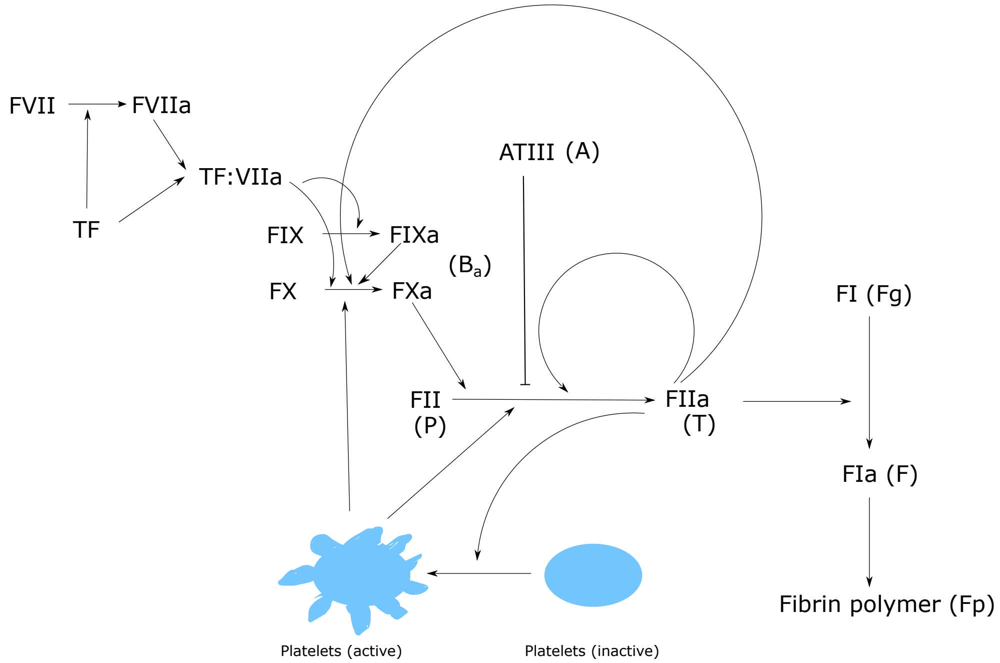

Fig 1. Schematic representation of the part of the coagulation cascade described in the model

$$\frac{\partial P}{\partial t} + \nabla \cdot (\mathbf{u}P - D\nabla P) = -\left(k_1\phi_c + k_2 B_a + k_3 T + k_4 T^2 + k_5 T^3\right) P, \tag{1}$$

here and in what follows, $\mathbf{u}$ is the flow velocity and D is the diffusion coefficient taken the same for all the clotting factors. The term in the right-hand side of the equation represents the activation of prothrombin by the active factors present on the surface of active platelets, factors IXa and Xa, and factors of the propagation phase respectively. Thrombin activates clotting factors of the propagation phase such as FV, FXIII, and FXI. Thus, the concentration of their active counterparts is proportional to thrombin concentration. The mathematical derivation of the polynomial terms describing the self-amplifying production of thrombin and their exact expressions are presented in the Appendix A. The rate of prothrombin consumption is equal to the rate of thrombin generation. Hence, we describe the concentration of thrombin as follows:

$$\frac{\partial T}{\partial t} + \nabla \cdot (\mathbf{u}T - D\nabla T) = \left(k_1\phi_c + k_2 B_a + k_3 T + k_4 T^2 + k_5 T^3\right) P - k_6 A T, \tag{2}$$

where the last term $-k_6 AT$ represents the inhibition of thrombin by prothrombin. Next, we describe the concentration of the clotting factors FIXa and FXa involved in the initiation phase. We denote their concentrations by B_a:

$$\frac{\partial B_a}{\partial t} + \nabla \cdot (\mathbf{u}B_a - D\nabla B_a) = k_7\phi_c(B^0 - B_a) + k_8 T(B^0 - B_a) - k_9 A B_a, \tag{3}$$

where the term in the right-hand side of the equation represents the activation of FIX and FX, $B = B^0 - B_a$ by active platelets. The second term describes their activation by thrombin and the last term accounts for their inhibition by antithrombin. We will present the boundary conditions for B_a below. Now, let us describe the concentration of antithrombin:

$$\frac{\partial A}{\partial t} + \nabla \cdot (\mathbf{u}A - D\nabla A) = -k_6 A T - k_9 A B_a, \tag{4}$$

where the first and second terms in the right-hand side of the equation represent the antithrombin consumption during its inhibition of thrombin as well as factors IXa and Xa. Next, we describe the concentration of fibrinogen F_g:

$$\frac{\partial F_g}{\partial t} + \nabla \cdot (\mathbf{u}F_g - D\nabla F_g) = -\frac{k_{10} T F_g}{K_{10} + F_g}, \tag{5}$$

where the term $-\frac{k_{10} T F_g}{K_{10}+F_g}$ represents the rate of fibrinogen conversion to fibrin by thrombin. This rate of fibrinogen consumption is equal to the production rate of fibrin. Hence, we describe the concentration of fibrin as follows:

$$\frac{\partial F}{\partial t} + \nabla \cdot (\mathbf{u}F - D\nabla F) = \frac{k_{10} T F_g}{K_{10} + F_g} - k_{11} F, \tag{6}$$

where the last term in the right-hand side of the equation represents the conversion of fibrin into fibrin polymer. Fibrin polymer concentration is described as follows:

$$\frac{\partial F_p}{\partial t} = k_{11} F. \tag{7}$$

Fibrin polymer forms a solid clot. Hence, it does not diffuse and it is not transported by flow. Next, we describe the density of platelets in flow ϕ_f and in the clot ϕ_c:

$$\frac{\partial \phi_f}{\partial t} + \nabla \cdot k(\phi_c + \phi_f)\left(\mathbf{u}\phi_f - D_p \nabla \phi_f\right) = -k_{12} T \phi_f - k_{13}\phi_f \phi_c, \tag{8}$$

$$\frac{\partial \phi_c}{\partial t} + \nabla \cdot k(\phi_c + \phi_f)\,(\mathbf{u}\phi_c - D_p \nabla \phi_c) = k_{12} T \phi_f + k_{13} \phi_f \phi_c, \tag{9}$$

where $k(\phi_c + \phi_f)D_p$ is the effective diffusion coefficient for platelets, $k(\phi_c + \phi_f)$ is a decreasing function given by $k(\phi_c + \phi_f) = \tanh\left(\pi\left(1 - \frac{\phi_c + \phi_f}{\phi_{max}}\right)\right)$. The behaviour of platelets in the flow depends on concentration of platelets [22] and resembles that of traffic flow. We take into account that thrombin activates platelets in the flow and contributes to their attachment to the clot. These platelets can also become active if they get into contact with the active platelets in the clot.

2.1.2 Boundary conditions

We apply the zero-flux condition at the all boundaries for thrombin, fibrin, and fibrin polymer. The concentrations of prothrombin, antithrombin, and fibrinogen were assumed to be at the physiological level in the plasma at the inlet. We set the concentration of platelets in the flow to $\phi_f = \phi_f^0$ as the initial condition and at the influx boundary. We apply the condition $\phi_c = \phi_c^0$ at the injured area to represent the initial concentration of active platelets bound to the subendothelium and the zero-flux boundary condition at the rest of the boundary.

In order to describe the generation of thrombin in the initiation phase at the damaged endothelial wall, we consider the complex T_F^* formed by the tissue factor and factor VII. Factors IX and X interact with this complex due to a surface reaction. They come from the bulk solution being inactive, form a complex $[T_F^* B]$ with T_F^* and return to the solution in the active form. The reaction rate for the surface concentration of this complex can be written as follows:

$$D\frac{\partial [T_F^* B]}{\partial \mathbf{n}} = k_f^+ B\,(T_F^* - [T_F^* B]) - k_f^- [T_F^* B],$$

where the first term in the right-hand side of this equation describes the flux of B to the surface, the second term describes the flux from the surface. Assuming that this reaction is fast, we can use the detailed equilibrium:

$$k_f^+ B\,(T_F^* - [T_F^* B]) = k_f^- [T_F^* B].$$

Then

$$D[T_F^* B] = \frac{k_f B T_F^*}{1 + k_f B}, \tag{10}$$

where $k_f = k_f^+ / k_f^-$.

The boundary conditions for the variables B and B_a at the damaged surface are as follows:

$$D\frac{\partial B}{\partial \mathbf{n}} = -k_f^+ B(T_F^* - [T_F^* B]), \quad D\frac{\partial B_a}{\partial \mathbf{n}} = k_f^- [T_F^* B]. \tag{11}$$

We prescribe the zero flux condition at the intact surface.

Assuming that the sum of the concentrations of activated and inactivated factors remains approximately constant because the rate of factors IXa and Xa consumption by antithrombin is very low, $B_a + B = B^0$, we obtain from (10) and the second equality in (11):

$$\frac{\partial B_a}{\partial \mathbf{n}}\Big|_{\Gamma_d} = \frac{\alpha_1\left(B^0 - B_a\right)}{D\left(1 + \beta_1 (B^0 - B_a)\right)}, \tag{12}$$

where $\alpha_1 = k_f^- k_f T_F^*$ and $\beta_1 = k_f$.

In the case of nonzero flow velocity, the concentrations of prothrombin, fibrinogen and antithrombin at the entrance of the domain $\mathbf{x} = 0$ are kept constant.

2.1.3 Blood flow model

We consider blood plasma as an incompressible Newtonian fluid and use the Navier-Stokes equations to describe the dynamics of blood flow in the vessel with rigid walls and its influence on clot growth dynamics:

$$\begin{aligned} \rho\frac{\partial \mathbf{u}}{\partial t} + \operatorname{div}\left(\rho \mathbf{u}\mathbf{u}^T - \mu\nabla\mathbf{u} + Ip\right) &= -\frac{\mu}{K_f}\mathbf{u}, \\ \operatorname{div}\left(\mathbf{u}\right) &= 0, \end{aligned} \tag{13}$$

where $\mathbf{u}$ is the flow velocity contributing to (1)-(9), p is the pressure, ρ is the density of the blood, μ is the dynamic viscosity, K_f is the hydraulic permeability of the clot [6]:

$$\frac{1}{K_f} = \frac{16}{\alpha^2}\widetilde{F}_p^{\frac{3}{2}}\left(1 + 56\widetilde{F}_p^3\right)\left(\frac{\phi_{max} + \phi_c}{\phi_{max} - \phi_c}\right), \tag{14}$$

here $\widetilde{F_p} = \min\left(\frac{7}{10}, \frac{F_p}{7000}\right)$ is the normalized concentration of fibrin polymer in the clot, α is the radius of the fibers.

Blood flow is driven by the pressure difference, we prescribe the pressure p_{in} at the inlet Γ_{in} and the pressure p_{out} at the outlet Γ_{out} and no-slip boundary condition $\mathbf{u} = \mathbf{0}$ at the rest of the boundary $\partial\Omega \setminus (\Gamma_{in} \cup \Gamma_{out})$. To set the inlet pressure in dependence on shear rate parameter γ, we use the formula $p_{in} = 4L\gamma\mu/D$, where L is the length of the vessel and D is the diameter of the vessel. The outflow pressure is set to zero $p_{out} = 0$. Note that red blood cells (RBCs) and their effects are not considered in the present work because the microfluidic experiments that are used to validate the model are performed on blood plasma which does not contain these cells. Contrary to the condition of fixed inlet velocity, the imposed pressure condition allows the clot to completely occlude the vessel and obstruct the flow.

2.1.4 Numerical implementation of the model

The computational grid for a vessel of 8 mm length and 1 mm diameter is shown in Figure 2. The TF patch, with the width 0.2 mm, is located at 2 mm from the vessel inlet. The grid consists of 20160 hexahedral cells and it is refined towards the surface of the cylinder (Figure 2, B). The refinement is required for more accurate recovery of the distribution of platelets in the vicinity of the vessel wall and the fibrin polymer formation.

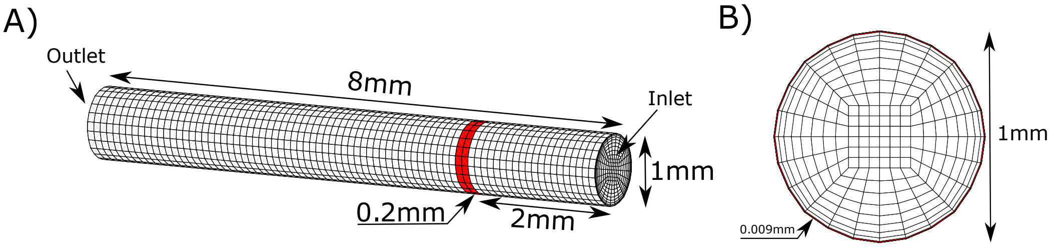

Fig 2. A) The settings for 3D numerical simulations of the coagulation process. Red strip at the surface indicates the TF patch with the width of 0.2 mm. B) Cross-section of the computational domain. The mesh is refined towards the vessel wall. The width of the numerical cells at the boundary is approximately 0.009 mm.

We use a novel cell-centered finite volume solver with a collocated arrangement of the velocity, pressure and all the clotting factors. The discrete problem is solved in a fully-coupled fully-implicit manner using finite volume discretizations.

The equations (13) may be rewritten in the form div $(\mathbf{A}) = \mathbf{g}$ where $\mathbf{A}$ is the flux depending on $\mathbf{u}$ and p. Integrating over each cell ω of the computational mesh and applying Green-Gauss theorem we get

$$\int_{\omega} \mathrm{div}\,(\mathbf{A})\,\mathrm{d}\omega = \oint_{\partial\omega} \mathbf{A}\cdot\mathbf{n}\mathrm{d}S = \int_{\omega} \mathbf{g}\mathrm{d}\omega$$

which is reformulated in terms of the vector flux $\mathbf{t} = \mathbf{A}\cdot\mathbf{n}$

$$\sum_{\text{faces } f \text{ of cell } \omega} \mathbf{t}|f| = \mathbf{g}|\omega|. \tag{15}$$

Equations (13) and vector representation of unknowns $(\mathbf{u}, p)^T$ yield decomposition of $\mathbf{t}$

$$\mathbf{t} = \begin{pmatrix} \rho\mathbf{u}\mathbf{u}^T\mathbf{n} - \mu\nabla\mathbf{u}\mathbf{n} + p\mathbf{n} \\ \mathbf{n}^T\mathbf{u} \end{pmatrix} = \begin{pmatrix} \rho\mathbf{u}\mathbf{u}^T\mathbf{n} \\ 0 \end{pmatrix} - \begin{pmatrix} \mu\nabla\mathbf{u}\mathbf{n} \\ 0 \end{pmatrix} + \begin{pmatrix} 0 & \mathbf{n} \\ \mathbf{n}^T & 0 \end{pmatrix}\begin{pmatrix} \mathbf{u} \\ p \end{pmatrix}. \tag{16}$$

Plugging discretizations of each term in (16) into the cell balance equation (15) defines the finite volume scheme. The details of the discretization are given elsewhere [30]. The method is stable, second-order accurate and has a compact computational stencil composed of cells neighbouring through face. The accuracy of the scheme was verified by comparisons with the analytical solutions of the Ethier-Steinman [31] and Poiseuille flows. Advection-diffusion-reaction equations (1)-(9) are discretized by a positivity preserving cell-centered finite volume scheme [32, 33] which in our case has the first-order accuracy due to the single point upstream approximation of the advective flux. The finite volume scheme for platelets equations (8),(9) use more elaborated stable flux discretization. The simulation was performed on INM RAS cluster. The tools for automatic differentiation, parallel mesh management, parallel linear and nonlinear solvers are provided by the INMOST library [34]. More details of the numerical schemes discretizing equations (1)-(9) and the simulation are given in [30], computational issues are beyond the scope of the present paper.

2.2 Thrombin generation model

Thrombin generation tests are widely used for the evaluation of blood coagulation in individual plasma samples. These tests measure the concentration of generated thrombin over time after the introduction of an amount of TF to plasma. We suggest a new model for thrombin generation in the presence of platelets in the motionless plasma. We first describe the concentration of FXa, $[Xa]$ using the following equation:

$$\begin{aligned}\frac{d[Xa]}{dt} &= a_1 TF[VIIa]([X]_0 - [Xa]) + a_2[IIa]([X]_0 - [Xa]) \\ &\quad + a_3\phi_0[IIa]([X]_0 - [Xa]) - a_4[Xa][ATIII],\end{aligned} \tag{17}$$

where the TF and $[VIIa]$ denote the concentrations of tissue factor and FVIIa respectively. The first term in the right-hand side of this equation represents the activation of FX by the complex TF-FVIIa. The second term describes the activation of FX by thrombin. The third term gives the activation rate of FX by platelets whose activation, in its turn, depends on thrombin. The last term describes the inhibition of FXa by antithrombin $[ATIII]$.

Next, we describe the concentration of prothrombin $[II]$:

$$\frac{d[II]}{dt} = -\left(b_1[Xa] + k_3[IIa] + k_4[IIa]^2 + k_5[IIa]^3\right)[II], \tag{18}$$

where the right-hand side describes the consumption of prothrombin upon its conversion to thrombin. Prothrombin is activated by FXa in the initiation phase and by other factors, such as FV, FVIII, and FXI in the propagation phase. These factors are activated by thrombin $[II]$. Therefore, the density of their active form is proportional to thrombin concentration. We describe the concentration of thrombin $[IIa]$ by the following equation:

$$\frac{d[IIa]}{dt} = \left(b_1[Xa] + k_3[IIa] + k_4[IIa]^2 + k_5[IIa]^3\right)[II] - b_2[ATIII][IIa]. \tag{19}$$

The rate of thrombin production is equal to the consumption rate of prothrombin. The second term in the right-hand side of this equation represents the direct inhibition of thrombin by antithrombin. The concentration of the latter $[ATIII]$ is described as follows:

$$\frac{d[ATIII]}{dt} = -a_4[Xa][ATIII] - b_2[IIa][ATIII]. \tag{20}$$

Initial concentrations of blood factors and the values of reaction rate constants are given in Table 1 in the Appendix B. The reaction rates in the model (17)-(20) (without platelets) are chosen by fitting thrombin generation curves obtained in a more complete model [14] with known parameters. Then, the parameter a_3 was fitted to reproduced the experimental results.

2.3 A simplified model of thrombin distribution

Thrombin plays an important role in the coagulation process as it converts fibrinogen into fibrin and then to fibrin polymer forming the clot. Similarly to our previous works [26, 27], we derive the following simplified 1D model for thrombin distribution from the spatiotemporal model of thrombus formation presented in Section 2.1:

$$\frac{\partial T}{\partial t} = D\frac{\partial^2 T}{\partial y^2} + \Phi(T, y), \tag{21}$$

where

$$\Phi(T, y) = \left(k_1\phi_0 + k_2B_a(y) + k_3T + k_4T^2 + k_5T^3\right)(P_0 - T) - \sigma(y)T,$$

D is the diffusion coefficient, P_0 is the prothrombin concentration initially present in blood flow, k_1, k_2, k_3, k_4, and k_5 are kinetic coefficients. The function $\sigma(y) = k_6A_0 + a\gamma$ shows the influence of antithrombin A_0 and of the blood flow in the downregulation of thrombin production. Here γ is the shear rate. Various simplifying hypotheses were considered in the derivation of the model. First, we suppose that consumption of clotting factors such as prothrombin and antithrombin does not significantly affect thrombus formation. Next, we assume that clot growth does not affect the local hemodynamics, and that the latter exerts a constant shear rate during the whole process of blood coagulation. Furthermore, we suppose that $B_a(y)$ is the stationary solution of equation (3). To simplify the interpretation of the model, we do not consider the activation of $B_a(y)$ by T and ϕ_0 at this stage. The general solution is then given by the form: $B(y) = B^0e^{-\sigma y}$. The production of thrombin by platelets and its amplification are directly considered in the thrombin equation. Note that the simplified 1D model (21) exhibits the same dynamics and the same speed of thrombin propagation as the more complete model [27–29]. Hence, the model approximates the evolution of the maximal height reached by the distribution of thrombin over time. Therefore, it provides a tool to describe the rate of vessel occlusion.

2.4 Identification of the parameters

We use a data-driven approach to calibrate the parameters of the models. First, we consider coagulation kinetics in the absence of platelets. The reaction rate constants for this system were taken from the literature except for the parameters k_3, k_4, and k_5 which are specific to the model. These contants can be written as a function of the activation and inhibition rates of the reactions that participate in the amplification of thrombin production (Appendix A). They were identified using the thrombin generation model by comparing with data in reference [14] using a Monte-Carlo algorithm to speed up the process. Then, we fitted the values of physiological parameters (blood clotting concentrations) to reproduce the experimental results describing the initiation time for NPP [11]. The rate of thrombin activation by platelets was fitted by comparing the output of the simplified model with the experimental data for the PRP [11], while the reaction constants for platelet dynamics k_{12} and k_{13} were taken from the literature [22,35]. The values of the parameters used in the model of thrombus formation in flow are provided in Table 2 in the Appendix B.

3 Numerical results

3.1 Quantification of the relationship between platelet count and blood coagulability

In order to evaluate the relationship between platelet count and blood coagulability, we use the thrombin generation model from Section 2.2 to reproduce the experiment conducted in reference [36]. These experiments consist of monitoring the generation of thrombin in mice blood with different platelet count after the introduction of 1 pM of TF. Thrombin generation curves can be characterized by different values such as the peak value and the endogenous thrombin potential (ETP) which corresponds to the total value of generated thrombin over time. The study shows the existence of a linear relationship between platelet count and the measured peak thrombin value. A hyperbolic relationship was also observed between platelet count and the ETP value. The model of thrombin generation was able to successfully reproduce these results as shown in Figure 3. Indeed, the similarity between the simulated and observed peak value and ETP implies that the thrombin generation curves are in a good agreement as well. We speculate that the small differences between the measured values and the predicted ones can be due to the intersubject variability in such experiments. In blood coagulation, the propagation phase is triggered when the generated value of thrombin reaches a certain threshold. The model predicts that the peak concentration of generated thrombin reaches 141.4 nM when the platelet count corresponds to $350 \times 10^9 L^{-1}$. This value is sufficient to provoke thrombin wave propagation according to the simplified model (equation (21)).

3.2 The shear rate threshold that prevents clotting initiation depends on the density of platelets

To investigate the dynamics that characterizes the initiation of clotting in blood flow, we use the model of thrombus growth (1)-(14) to reproduce the experiment on *in vitro* clot growth [11]. In this experiment, blood plasma can be described by a Newtonian flow driven by a constant pressure drop. The computational domain corresponds to the tube with rigid walls. We run numerical simulations for a physical time corresponding to 1200 s and determine the initiation time of blood coagulation corresponding to the beginning of the propagation phase when a sudden elevation of the maximal value of

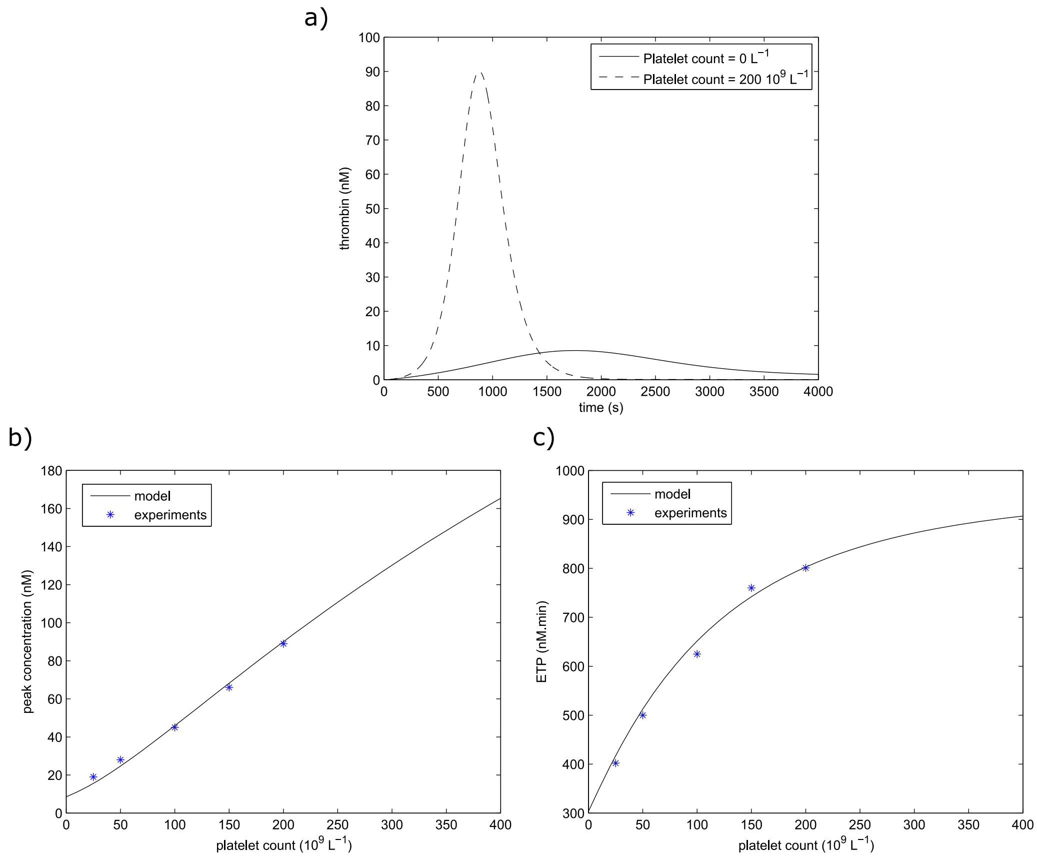

Fig 3. a) Thrombin generation curves for two values of platelet count. b) The relationship between thrombin peak value and platelet count according to model predictions and experiments. c) The relationship between ETP and platelet count as reported by the model and experiments.

generated fibrin polymer near the TF patch can be observed. This moment coincides with the time when the vessel is no longer completely permeable and the clot will subsequently occlude the vessel. We vary the value of the pressure drop to control the initial shear rate. Then, the value of the shear rate can also change if clot growth is initiated. However, in this case we intend to determine the value of the shear rate that prevents the initiation of the coagulation process. Therefore, we determine the value of the initial shear rate that prevents blood clotting by running consecutive simulations. We consider that for PRP, platelet density is equal to $300 \times 10^9\ L^{-1}$ which is the value considered in the experiments [11]. Similarly to the findings of the experiments, a threshold response of clotting initiation to the shear rate was observed in the numerical simulations. When the shear rate is lower than a threshold value, coagulation is initiated at an early time before $t = 800\ s$ and the thrombus occludes the vessel. In this case, blood flow can be completely stopped and the stagnant plasma turns into a thrombus. When the value of the shear rate is sufficiently high, the initiation of the coagulation process takes a longer time (higher than $t = 800\ s$). Figure 4 and Figure 5 represent the results of numerical simulations of thrombus formation for platelet-rich plasma at the shear rate $\gamma = 25\ s^{-1}$ and times $t = 60\ s$ and $t = 70\ s$, respectively. For illustration purposes, the velocity magnitude is demonstrated in the log-scale. From the velocity distribution, it is evident that the flow is obstructed by the clot at time $t = 70\ s$. Both figures show that the model is symmetrical with respect to the azimuth

angle, but not with regard to the height of the cylinder.

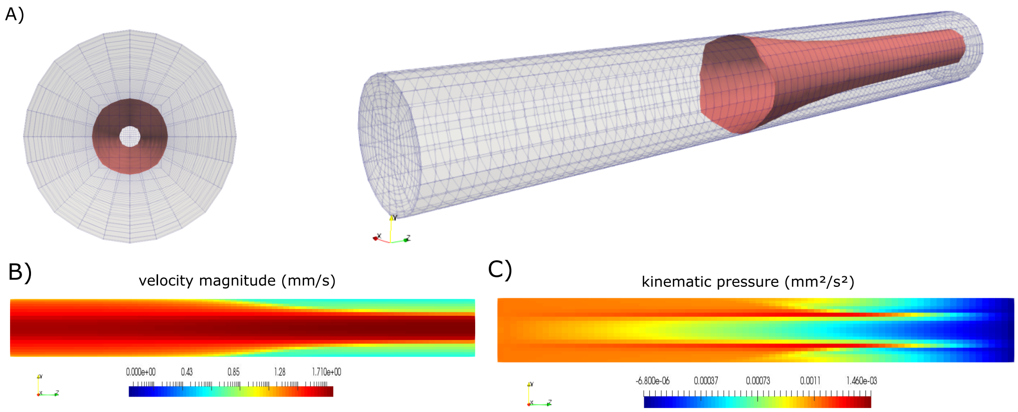

Fig 4. Fluid dynamics before vessel occlusion for platelet-rich plasma (PRP) for the shear rate $\gamma = 25\ s^{-1}$ and at the time $t = 60\ s$. The vessel is still permeable, although the flow is significantly obstructed by the clot. A) isosurface of fibrin polymer corresponding to the hydraulic resistance $1/K_f = 10\ mm^{-2}$. B) The magnitude of velocity in the log-scale. C) The distribution of pressure.

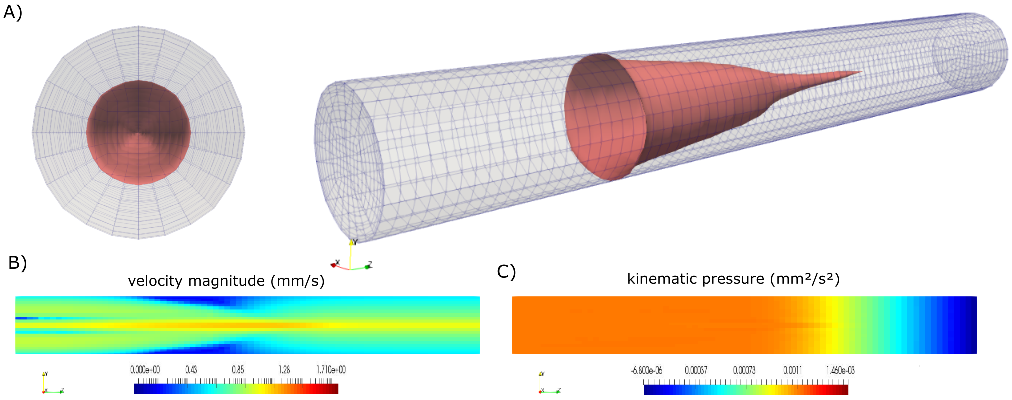

Fig 5. Fluid dynamics after the occlusion of the vessel for platelet rich-plasma for the shear rate $\gamma = 25\ s^{-1}$ and at the time $t = 70\ s$. The vessel is already impermeable due to the clot. A) isosurface of fibrin polymer corresponding to the hydraulic resistance $1/K_f = 10\ mm^{-2}$. B) The magnitude of velocity in the log-scale. C) The distribution of pressure.

In order to compare the simulation results with the experiments in the *in vitro* microfluidic chambers [11], we compute the necessary time for blood clotting initiation while increasing the shear rate γ. A threshold response is observed in the initiation of blood clotting characterized by a critical value which is higher in platelets rich plasma (Figure 6, A). We observed that the value of the clotting initiation time becomes stable for high shear rates in long tubes. However, it keeps increasing exponentially for short tubes. The simplified model predicts a sharp transition between the regimes of complete and partial occlusion when the shear rate exceeds the threshold value (Figure 6, B). These critical values are in good agreement with the ones reported in the experiments (Figure 6, A) and with the results of the full 3D model (Figure 6, C).

While numerical simulations can be used to have a realistic representation of the process of clot growth, the simplified model for thrombin distribution (21) provides a

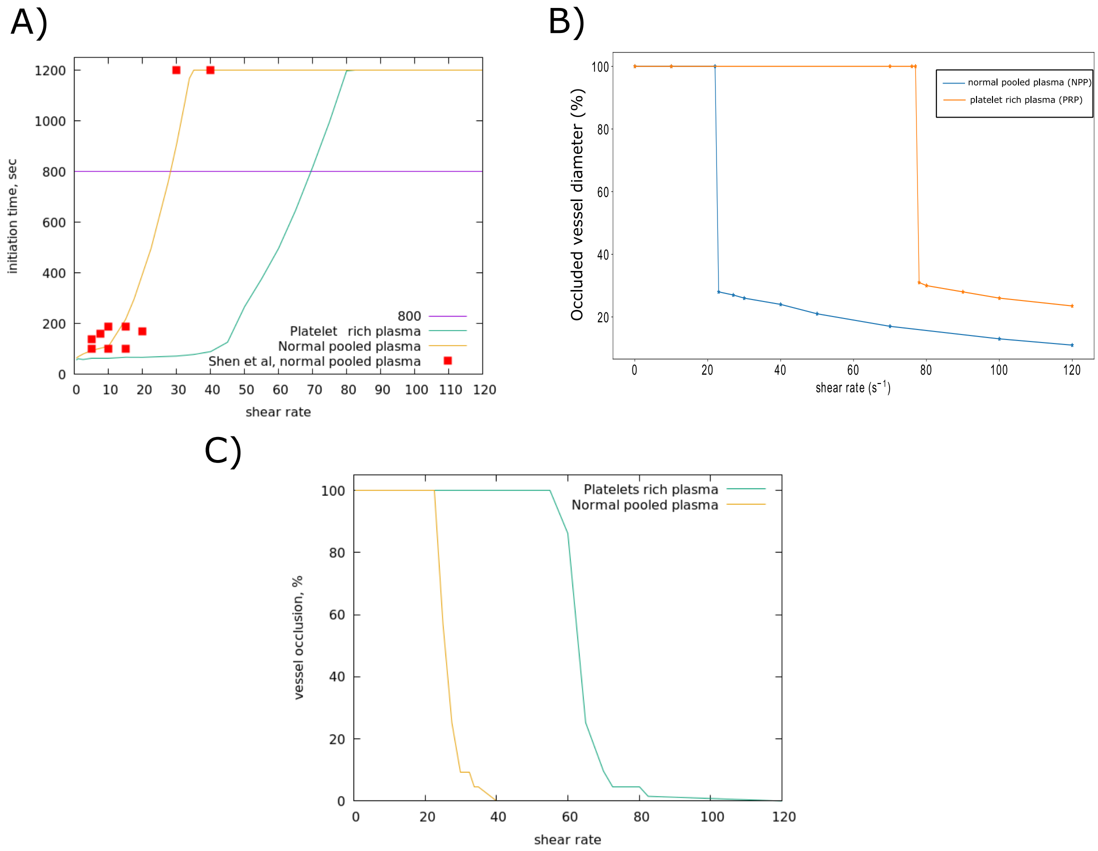

Fig 6. A) The moment of clotting initiation as a function of the initial wall shear rate for NPP and PRP for a long tube with the length 8 mm. Experimental results of *in vitro* thrombus formation are shown for comparison purposes (reproduced from [11, 37]). B) The percentage of the vessel occupied by the clot at $t = 800$ s according to the one-equation 1D model. Occluded regions are domains where teh concentration of thrombin exceeds 200 nM. C) The percentage of the occluded vessel at $t = 800$ s according to the full 3D model.

powerful tool to analyze the various mechanisms involved in the development of venous thrombi. The production of thrombin is followed by the formation of the fibrin mesh. Therefore, we consider that the percent of the vessel that is occluded corresponds to the regions where thrombin exceeds a certain threshold $T^* = 200$ nM. For equation (21), it is known that the wave speed is positive if and only if

$$\int_{T_0}^{T_2} \Phi(T, y) dT > 0. \tag{22}$$

Here T_0 and T_2 are stable zeros of the function $\Phi(T, y)$. Note that this condition is verified for a fixed y assuming that the reaction front is narrow. This condition also gives the clot size which corresponds to the value of y where the clot stops growing. The condition on shear rate for thrombin propagation is as follows:

$$\gamma > \gamma^*, \tag{23}$$

where

$$\gamma^* = \frac{1}{aT_2/2}\begin{pmatrix} k_1\left(\phi_0 P_0 T_2/2 - \phi_0 T_2^2/3\right) + k_2\left(B_a(y)P_0 - B_a(y)T_2/2\right) + k_3\left(P_0 T_2^1/2 - T_2^2/3\right) + \\ k_4\left(P_0 T_2^4/3 - T_2^3/4\right) + k_5\left(P_0 T_2^3/4 - T_2^4/5\right) - k_6 A_0 T_2/2 - av(y)T_2/2 \end{pmatrix}.$$

The threshold of shear rate that initiates the coagulation process, is linearly proportional to the platelet density for various values of prothrombin and antithrombin concentrations as shown in Figure 7. The concentration of prothrombin has a greater impact on the this linear relationship than the concentration of antithrombin.

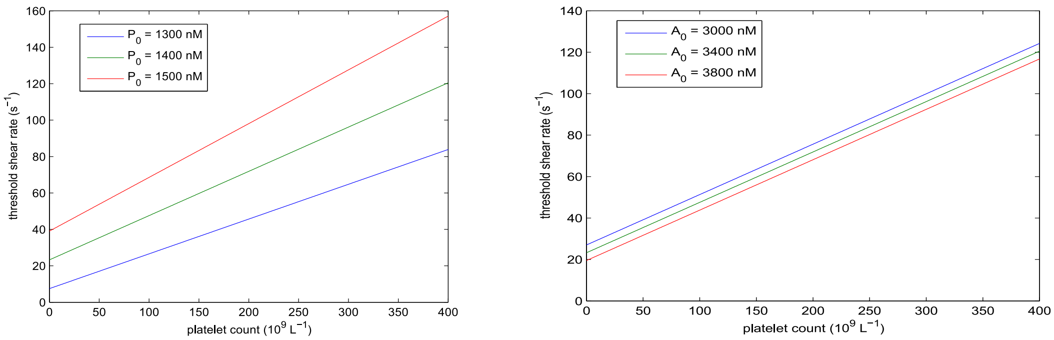

Fig 7. The shear rate threshold of clotting initiation as a function of platelets density for different prothrombin (left) and antithrombin concentrations (right).

3.3 Quantification of the effect of TF surface area on the initiation of blood clotting

The surface of exposed TF is another important factor that determines the necessary time for blood clotting initiation. To explore the effects of this parameter on the coagulation process, we conduct systematic numerical simulations for different sizes of the TF patch with NPP. We impose a fixed shear rate equal to $\gamma = 40\ s^{-1}$ in all these simulations. Increasing the size of the TF patch increases the production of FIXa and FXa which upregulates the generation of thrombin and accelerates the clotting initiation. As expected, numerical simulations show the existence of a threshold value for the TF patch that initiates blood coagulation (Figure 8). As before, the value above which the initiation of blood clotting can be observed is very close to the one observed in experiments. This threshold response to the size of the TF patch was also observed in other experimental studies [38, 39].

4 Discussion

This work presents a quantitative study of the effects of platelet count, wall shear rate, and injury size on the initiation of blood coagulation based on the analysis of the model and on numerical simulations. The calibration of previously developed models allowed us to describe the results of clinical studies and *in vitro* experiments in microfluidic chambers. To evaluate the effects of platelets on blood coagulability, we have simulated the thrombin generation curves for blood samples with variable platelet counts. Similarly to the experiments [36], a linear relation was observed between the platelet count and the peak value reached by thrombin concentration. Furthermore, the existence of a positive hyperbolic relation between the platelet count and the ETP was confirmed. These relationships are in good agreement with the clinical indications that

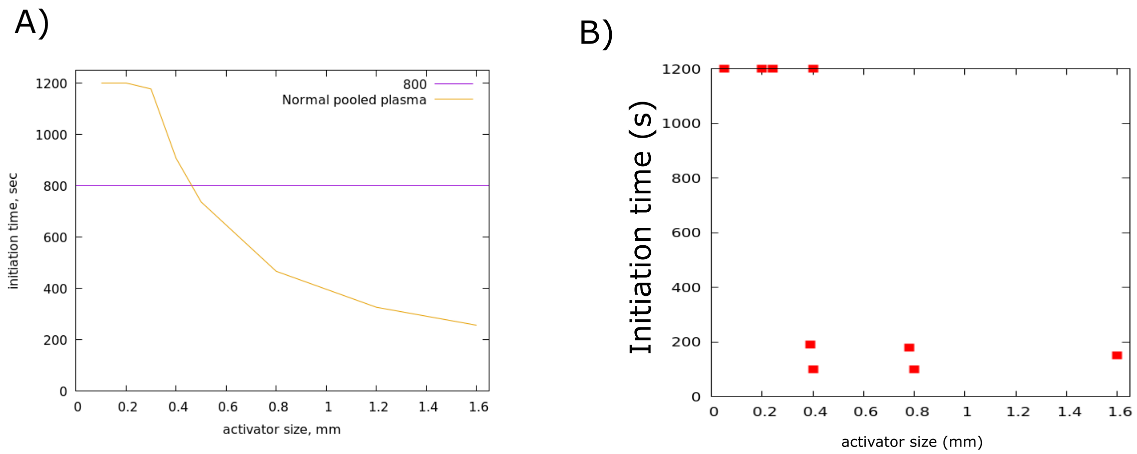

Fig 8. A) The NPP and PRP clotting initiation time as a function of the TF patch size for a constant shear rate equal to 40 s^{-1}. B) Experimental data showing the time of clotting initiation for different sizes of the activator [11].

consider a high platelet count as an independent risk factor for VTE while a low platelet count (thrombocytopenia) is considered to be associated with hemorrhagic events [40].

Hypercoagulability caused by high platelet count also accelerates the process of thrombus formation which increases the risk of venous thrombosis incidence. To explore the conditions that trigger venous thrombosis, we used a previously developed model of blood coagulation to reproduce a set of experiments of thrombus growth in microfluidic capillaries [11]. A novel 3D finite-volume solver was developed and used to mimic the settings of these experiments. The plasma was described as a Newtonian fluid driven by a constant non-pulsative pressure difference. Similarly to the experiments, two types of plasma were considered: normal-pooled (or platelet poor) plasma (NPP) and platelet-rich plasma (PRP). The most important finding of these experiments is confirming that the initiation of coagulation displays a threshold response to shear rate. This was also observed in our numerical simulations and was accurately reproduced by choosing the appropriate physiological parameters. As expected, the shear rate value that is necessary to prevent the initiation of thrombus growth was higher in PRP. We speculate that for a constant pressure drop, decreasing the length of the vessel or increasing its diameter result in an increase of the shear rate that prevents clotting initiation. Using a simplified model, we were able to specify this value for a wide range of platelet count values as well as for large sets of physiological parameters. A similar threshold response of the clotting initiation was also observed for the size of the TF patch. This phenomenon was already investigated in another computational modelling study [41]. Another important result obtained both in modelling and in the *in vitro* experiments was that the initiation of blood coagulation depends on shear rate and not on blood flow velocity (i.e. linearly related to blood flow velocity divided by the diameter of the blood vessel).

In the present study, we used data-driven modelling to quantify the effects of platelet count, shear rate, and other pathophysiological parameters on the pathogenesis of venous thrombosis. The synergy between the presented models allowed us to study the combined effects of various interlinked factors of venous thromboembolism such as blood coagulability, hemodynamics, and the size of the TF patch. These aspects represent the three elements of the Virchow's triad [10]. We have conducted experimentally validated numerical simulations of thrombin generation and thrombus development in the flow. At the same time, we have used the one-equation model of thrombin distribution to derive analytical estimates of the shear rate threshold which prevents clotting initiation

for different plasma compositions. This model reduction replaced the parameter sensitivity analysis of the model of thrombus formation under the flow, which would require a much higher computational cost. Indeed, the main feature of the presented model in comparison with the rest of spatio-temporal models is the possibility to derive from it a thrombin generation model and a 1-D thrombin distribution model. As a result, it was possible to simulate the development of the thrombus under flow, to estimate the coagulability of blood, and to derive analytical estimates for the relationships between the initiation of coagulation and the various model parameters. Furthermore, there exist very few 3D continuous models that describe hemodynamics, the coagulation biochemistry, and platelet kinetics at the same time.. The combination of the three models was also essential for the identification of parameters. For example, by fitting the thrombin generation model with the experimental curves, we could determine the parameters that describe the propagation phase in the two other models.

In order to reproduce experiments, some simplifying assumptions were imposed. They include the Newtonian nature of flow (blood plasma in this case), the non-pulsatile continuous aspect of flow, and the rigidity of the vessel wall (microfluidic capillaries). The similarity between the modelling conditions and these settings not only gave credibility to the quantitative predictions of the model but also allowed us to calibrate the model and to use it for the upcoming studies. The effects of RBCs can be taken into consideration implicitly through the choice of blood rheological properties. Their participation in the coagulation process consists in marginalizing platelets to the vessel wall. Thus, they can accelerate the clotting process [42], but this will not change the qualitative predictions made by the model and experiments that consider blood plasma. In fact, the threshold response to the TF patch size was observed under blood flow that contains RBCs [39] as well. The marginalization of platelets by RBCs can be introduced implicitly by using discrete methods such as dissipative particle dynamics [43] and immersed boundary methods [44]. Furthemore, blood can be simulated as a multi-constituent fluid with one phase for the plasma with RBCs and the other phase representing the plasma with platelets [45]. Another limitation of this study concerns the origin of blood in the experimental studies that were used to calibrate the model. Human blood was considered in the microfluidic experiments of clotting initiation [11], whereas mice plasma was used in the experiments of thrombin generation [36]. The mice plasma contains approximately the same concentrations of blood factors as its human counterpart [46]. The major differences between the two are the density of platelets and the concentrations of anticoagulant factors. In the experiments [36], the concentrations of antithrombin and TFPI were reduced by diluting the plasma and the density of platelets was varied in order to quantify its impact on thrombin generation. In the forthcoming works, we will quantify the effects of different physical and pathophysiological parameters on clot formation in non-Newtonian, pulsatile flow and inside elastic blood vessels. Furthermore, we will also use the model to reproduce other microfluidic experiments corresponding to other blood clotting scenarios.

Acknowledgements

K.Terekhov was supported by RFBR grant 18-31-20048. Yu. Vassilevski was supported by the world-class research center "Moscow Center for Fundamental and Applied Mathematics". V. Volpert was supported by the "RUDN University Program 5-100."

Appendix A: derivation of the model

Let us consider the following model which describes the self-amplifying production of thrombin:

$$\frac{\partial[Va]}{\partial t} = D\Delta[Va] + \widehat{k}_1 T - h_1[Va], \tag{24}$$

$$\frac{\partial[VIIIa]}{\partial t} = D\Delta[VIIIa] + \widehat{k}_2 T - h_2[VIIIa], \tag{25}$$

$$\frac{\partial[XIa]}{\partial t} = D\Delta[XIa] + \widehat{k}_3 T - h_3[XIa], \tag{26}$$

$$\frac{\partial[IXa]}{\partial t} = D\Delta[IXa] + \widehat{k}_4[XIa] - h_4[IXa], \tag{27}$$

$$\frac{\partial[Xa]}{\partial t} = D\Delta[Xa] + \widehat{k}_5[IXa] + \widehat{k}_{55}[VIIIa][IXa] - h_5[Xa], \tag{28}$$

$$\frac{\partial T}{\partial t} = D\Delta T + \left(\widehat{k}_6[Xa] + \widehat{k}_{66}[Xa][Va]\right) P - \sigma T, \tag{29}$$

derived from another model of blood coagulation in plasma [47]. The coefficients $\widehat{k}_i$ denote the activation coefficient rates and h_i the inhibition rate. The factors Va and Xa form the prothrombinase complex Va-Xa while the factors VIIIa and IXa for the complex VIIIa-IXa. They are considered in (28) and (29) in the form of the terms $\widehat{k}_{55}[VIIIa][IXa]$ and $\widehat{k}_{66}[Xa][Va]$, which are obtained using the assumption of detailed equilibrium for large reaction constants. The model can be completed by adding the equations for platelets, prothrombin, antithrombin, fibrinogen, fibrin, and fibrin polymer. These equations do not influence the amplification of thrombin production and therefore the system (24)-(29) can be studied independently.

Under the assumption of detailed equilibrium, the concentrations of active coagulation factors can be expressed as follows:

$$\begin{gathered}[Va] = \frac{\widehat{k}_1}{h_1} T, \ [VIIIa] = \frac{\widehat{k}_2}{h_2} T, \ [XIa] = \frac{\widehat{k}_3}{h_3} T, \\ [IXa] = \frac{\widehat{k}_3\widehat{k}_4}{h_3 h_4} T, \ [Xa] = \frac{\widehat{k}_3\widehat{k}_4}{h_3 h_4} T \left(\frac{\widehat{k}_5}{h_5} + \frac{k_{55}\widehat{k}_2}{h_2 h_5} T \right). \end{gathered} \tag{30}$$

We substitute (30) in the equation of thrombin (29):

$$\frac{\partial T}{\partial t} = D\Delta T + \left(k_3 T + k_4 T^2 + k_5 T^3\right) P - \sigma T, \tag{31}$$

where

$$k_3 = \frac{\widehat{k}_3\widehat{k}_4\widehat{k}_5\widehat{k}_6}{h_3 h_4 h_5}, \quad k_4 = \frac{\widehat{k}_2\widehat{k}_3\widehat{k}_4\widehat{k}_{55}\widehat{k}_6}{h_2 h_3 h_4 h_5} + \frac{\widehat{k}_1\widehat{k}_3\widehat{k}_4\widehat{k}_5\widehat{k}_{66}}{h_1 h_3 h_4 h_5}, \quad k_5 = \frac{\widehat{k}_1\widehat{k}_2\widehat{k}_3\widehat{k}_4\widehat{k}_{55}\widehat{k}_{66}}{h_1 h_2 h_3 h_4 h_5}.$$

Equation (31) gives a good approximation of the clot growth rate described by the system (24)-(29) [48].

Table 1. Numerical values of the parameters used in the model of thrombin generation.

Simulation	Value	Unit	Description
dt	0.02	s	time step
Kinetic rates	Value	Unit	Reaction
a_1	1×10^{-5}	$nM^{-2}s^{-1}$	X activation by $TF - VII_a$
a_2	1×10^{-10}	$nM^{-1}s^{-1}$	X activation by $IIa - TF - VII$
a_3	1.34×10^{-2}	$nM^{-1}Ls^{-1}$	X activation by platelets
a_4	8×10^{-6}	$nM^{-1}s^{-1}$	X inactivation by antithrombin
a_1	1×10^{-5}	$nM^{-1}s^{-1}$	X activation by $TF - VII_a$
a_1	1×10^{-5}	$nM^{-1}s^{-1}$	X activation by $TF - VII_a$
b_1	3×10^{-2}	$nM^{-1}s^{-1}$	II activation by B_a
k_3	1.5×10^{-5}	$nM^{-1}s^{-1}$	II activation by T
k_4	8×10^{-6}	$nM^{-3}s^{-1}$	II activation by T^2
k_5	1×10^{-10}	$nM^{-3}s^{-1}$	II activation by T^3
b_2	1.5×6.7	$nM^{-6}s^{-1}$	IIa inactivation by antithrombin
Initial concentrations	Value	Unit	Description
TF	0.5×10^{-3}	nM	tissue factor concentrations
$[VIIa]$	10	nM	factor VIIa concentration
$[II]_0$	950	nM	initial prothrombin concentration
$[X]_0$	80	nM	initial FX concentration
$[ATIII]_0$	3000	nM	X initial antithrombin concentration

Table 2. Values of all parameters used in the model of thrombus formation in the flow.

Simulation	Value	Unit	Description
h	0.02	mm	space step
dt	0.005	s	time step
Diffusion coefficients	Value	Unit	
D	510^{-5}	mm^2/s	Blood factors diffusion coefficient [22]
D_p	2.510^{-5}	mm^2/s	Platelets diffusion coefficient [22]
Kinetic rates	Value	Unit	Reaction
k_1	7×10^{-5}	$10^{-9}Ls^{-1}$	P activation by ϕ_c (fitted)
k_2	7.5×10^{-6}	$nM^{-1}s^{-1}$	P activation by B_a [14]
k_3	1.5×10^{-5}	$nM^{-1}s^{-1}$	P activation by T (fitted)
k_4	8×10^{-6}	$nM^{-2}s^{-1}$	P activation by T^2 (fitted)
k_5	1×10^{-10}	$nM^{-3}s^{-1}$	P activation by T^3 (fitted)
k_6	4.817×10^{-6}	$nM^{-1}s^{-1}$	T inactivation by A [49]
k_7	1×10^{-9}	$10^{-9}Ls^{-1}$	B activation by ϕ_c (fitted)
k_8	5.2173×10^{-5}	$nM^{-1}s^{-1}$	B activation by T (estimated from [48])
k_9	2.223×10^{-9}	$nM^{-1}s^{-1}$	B_a inactivation by A [49]
k_{10}	0.05	s^{-1}	F_g activation by T [50]
K_{10}	3160	nM	substrate fibrin concentration [50]
k_{11}	0.1	s^{-1}	$F \to F_p$ fibrin polymerization
k_{12}	0.002	$nM^{-1}s^{-1}$	platelets activation by thrombin (estimated from [35])
k_{13}	4×10^{-9}	$10^{-9}Ls^{-1}$	platelets activation by platelets (estimated from [22])
Kinetic constants	Value	Unit	Description
α_1	7.7×10^4	$nM^{-1}s^{-1}$	$\alpha_1 = k_f^- k_f T_F^*$
β_1	0.225	nM^{-1}	$\beta_1 = k_f^+/k_f^-$
ϕ_{max}	400	10^9L^{-1}	maximal density of platelets
Kinetic concentrations	Value	Unit	Description
P_0	1400	nM	prothrombin concentration [14]
A_0	3400	nM	antithrombin concentration [14]
B^0	200	nM	sum of factors IX and X concentrations [14]
$[VII]$	10	nM	factor VII concentration [14]
Fg_0	7000	nM	fibrinogen concentration [14]
ϕ_f^0	300	10^9L^{-1}	platelets density [14]
ϕ_c^0	1	10^9L^{-1}	activated platelets bound to the subendothelium [14]
Flow properties	Value	Physical	Description
ρ	1.06×10^{-6}	$kgmm^{-3}$	plasma density
ν	1.3	$mm^{-2}s^{-1}$	blood plasma viscosity
α	6×10^{-4}	mm	fiber radius
a	5.6×1^{-3}	$s\ mm^{-1}$	thrombin removal rate by flow in the simplified model (fitted)

Appendix B: model parameters

References

1. Rasche H. Haemostasis and thrombosis: an overview. European Heart Journal Supplements. 2001;3(suppl_Q):Q3–Q7.

2. Bouchnita A, Volpert V. A multiscale model of platelet-fibrin thrombus growth in the flow. Computers & Fluids. 2019;.

3. Zakai N, Wright J, Cushman M. Risk factors for venous thrombosis in medical inpatients: validation of a thrombosis risk score. Journal of Thrombosis and Haemostasis. 2004;2(12):2156–2161.

4. Buss DH, Stuart JJ, Lipscomb GE. The incidence of thrombotic and hemorrhagic disorders in association with extreme thrombocytosis: an analysis of 129 cases. American journal of hematology. 1985;20(4):365–372.

5. Gerotziafas GT, Depasse F, Busson J, Leflem L, Elalamy I, Samama MM. Towards a standardization of thrombin generation assessment: the influence of tissue factor, platelets and phospholipids concentration on the normal values of Thrombogram-Thrombinoscope assay. Thrombosis journal. 2005;3(1):16.

6. Wufsus A, Macera N, Neeves K. The hydraulic permeability of blood clots as a function of fibrin and platelet density. Biophysical journal. 2013;104(8):1812–1823.

7. Belyaev AV. Catching platelets from the bloodflow: the role of the conformation of von Willebrand factor. Mathematical Modelling of Natural Phenomena. 2018;13(5):44.

8. Mann KG. Biochemistry and physiology of blood coagulation. Thrombosis and haemostasis. 1999;82(08):165–174.

9. Sambrano GR, Weiss EJ, Zheng YW, Huang W, Coughlin SR. Role of thrombin signalling in platelets in haemostasis and thrombosis. Nature. 2001;413(6851):74.

10. Brotman DJ, Deitcher SR, Lip GY, Matzdorff AC. Virchow's triad revisited. Southern medical journal. 2004;97(2):213–215.

11. Shen F, Kastrup CJ, Liu Y, Ismagilov RF. Threshold response of initiation of blood coagulation by tissue factor in patterned microfluidic capillaries is controlled by shear rate. Arteriosclerosis, thrombosis, and vascular biology. 2008;28(11):2035–2041.

12. Belyaev A, Dunster J, Gibbins J, Panteleev M, Volpert V. Modeling thrombosis in silico: Frontiers, challenges, unresolved problems and milestones. Physics of life reviews. 2018;26:57–95.

13. Fasano A, Sequeira A. Hemomath: The mathematics of blood. vol. 18. Springer; 2017.

14. Hockin MF, Jones KC, Everse SJ, Mann KG. A model for the stoichiometric regulation of blood coagulation. Journal of Biological Chemistry. 2002;277(21):18322–18333.

15. Anand M, Rajagopal K, Rajagopal K. A model for the formation, growth, and lysis of clots in quiescent plasma. A comparison between the effects of antithrombin III deficiency and protein C deficiency. Journal of theoretical biology. 2008;253(4):725–738.

16. Elizondo P, Fogelson AL. A mathematical model of venous thrombosis initiation. Biophysical journal. 2016;111(12):2722–2734.

17. Galochkina T, Chelushkin M, Sveshnikova A. Activation of Contact Pathway of Blood Coagulation on the Lipopolysaccharide Aggregates. Mathematical Modelling of Natural Phenomena. 2017;12(5):196–207.

18. Fogelson AL, Hussain YH, Leiderman K. Blood clot formation under flow: the importance of factor XI depends strongly on platelet count. Biophysical journal. 2012;102(1):10–18.

19. Tosenberger A, Salnikov V, Bessonov N, Babushkina E, Volpert V. Particle dynamics methods of blood flow simulations. Mathematical Modelling of Natural Phenomena. 2011;6(5):320–332.

20. Tosenberger A, Ataullakhanov F, Bessonov N, Panteleev M, Tokarev A, Volpert V. Modelling of platelet–fibrin clot formation in flow with a DPD–PDE method. Journal of mathematical biology. 2016;72(3):649–681.

21. Govindarajan V, Rakesh V, Reifman J, Mitrophanov AY. Computational study of thrombus formation and clotting factor effects under venous flow conditions. Biophysical journal. 2016;110(8):1869–1885.

22. Leiderman K, Fogelson AL. Grow with the flow: a spatial–temporal model of platelet deposition and blood coagulation under flow. Mathematical medicine and biology: a journal of the IMA. 2011;28(1):47–84.

23. Sequeira A, Bodnár T. Blood coagulation simulations using a viscoelastic model. Mathematical Modelling of Natural Phenomena. 2014;9(6):34–45.

24. Hosseinzadegan H, Tafti DK. Modeling thrombus formation and growth. Biotechnology and bioengineering. 2017;114(10):2154–2172.

25. Susree AM, Anand BM. Reaction mechanisms and kinetic constants used in mechanistic models of coagulation and fibrinolysis. Mathematical Modelling of Natural Phenomena. 2016;11(6):71–90.

26. Bouchnita A, Tosenberger A, Volpert V. On the regimes of blood coagulation. Applied Mathematics Letters. 2016;51:74–79.

27. Bouchnita A, Galochkina T, Kurbatova P, Nony P, Volpert V. Conditions of microvessel occlusion for blood coagulation in flow. International journal for numerical methods in biomedical engineering. 2017;33(9):e2850.

28. Bouchnita A, Miossec P, Tosenberger A, Volpert V. Modeling of the effects of IL-17 and TNF-α on endothelial cells and thrombus growth. Comptes rendus biologies. 2017;340(11-12):456–473.

29. Bouchnita A, Bouzaachane K, Galochkina T, Kurbatova P, Nony P, Volpert V. An individualized blood coagulation model to predict INR therapeutic range during warfarin treatment. Mathematical Modelling of Natural Phenomena. 2016;11(6):28–44.

30. Kapyrin I, Nikitin K, Terekhov K, Vassilevski Y. Parallel finite volume computation on general meshes. in production: Springer; 2019.

31. Ethier CR, Steinman D. Exact fully 3D Navier–Stokes solutions for benchmarking. International Journal for Numerical Methods in Fluids. 1994;19(5):369–375.

32. Lipnikov K, Svyatskiy D, Vassilevski Y. A monotone finite volume method for advection-diffusion equations on unstructured polygonal meshes. J Comput Phys. 2010;229:4017–4032.

33. Nikitin K, Vassilevski Y. A monotone nonlinear finite volume method for advection-diffusion equations on unstructured polyhedral meshes in 3D. Russian J Numer Anal Math Modelling. 2010;25(4):335–358.

34. Vassilevski Y, Konshin I, Kopytov G, KM T. INMOST - programming platform and graphical environment for development of parallel numerical models on general grids (in Russian). Moscow University Press. 2013; p. 144.

35. Kuharsky AL, Fogelson AL. Surface-mediated control of blood coagulation: the role of binding site densities and platelet deposition. Biophysical journal. 2001;80(3):1050–1074.

36. Dargaud Y, Spronk HM, Leenders P, Hemker HC, Ten Cate H. Monitoring platelet dependent thrombin generation in mice. Thrombosis research. 2010;126(5):436–441.

37. Shibeko A, Karamzin S, Butylin A, Panteleev M, Ataullakhanov F. The review of contemporary ideas about the influence of flow rate on blood clotting. Biochemistry (Moscow) Supplement Series A: Membrane and Cell Biology. 2009;3(4):388.

38. Kastrup CJ, Shen F, Runyon MK, Ismagilov RF. Characterization of the threshold response of initiation of blood clotting to stimulus patch size. Biophysical journal. 2007;93(8):2969–2977.

39. Okorie UM, Denney WS, Chatterjee MS, Neeves KB, Diamond SL. Determination of surface tissue factor thresholds that trigger coagulation at venous and arterial shear rates: amplification of 100 fM circulating tissue factor requires flow. Blood. 2008;111(7):3507–3513.

40. van der Bom JG, Heckbert SR, Lumley T, Holmes CE, Cushman M, Folsom AR, et al. Platelet count and the risk for thrombosis and death in the elderly. Journal of thrombosis and haemostasis. 2009;7(3):399–405.

41. Belyaev AV, Panteleev MA, Ataullakhanov FI. Threshold of microvascular occlusion: injury size defines the thrombosis scenario. Biophysical journal. 2015;109(2):450–456.

42. Litvinov RI, Weisel JW. Role of red blood cells in haemostasis and thrombosis. ISBT science series. 2017;12(1):176–183.

43. Tosenberger A, Salnikov V, Bessonov N, Babushkina E, Volpert V. Particle dynamics methods of blood flow simulations. Mathematical Modelling of Natural Phenomena. 2011;6(5):320–332.

44. Belyaev AV. Computer modelling of initial platelet adhesion during microvascular thrombosis. Russian Journal of Numerical Analysis and Mathematical Modelling. 2019;34(5):241–251.

45. Wu WT, Jamiolkowski MA, Wagner WR, Aubry N, Massoudi M, Antaki JF. Multi-constituent simulation of thrombus deposition. Scientific reports. 2017;7:42720.

46. Tsakiris DA, Scudder L, Hodivala-Dilke K, Hynes RO, Coller BS. Hemostasis in the mouse (Mus musculus): a review. Thrombosis and haemostasis. 1999;81(02):177–188.

47. Krasotkina YV, Sinauridze EI, Ataullakhanov FI. Spatiotemporal dynamics of fibrin formation and spreading of active thrombin entering non-recalcified plasma by diffusion. Biochimica et Biophysica Acta (BBA)-General Subjects. 2000;1474(3):337–345.

48. Galochkina T, Bouchnita A, Kurbatova P, Volpert V. Reaction-diffusion waves of blood coagulation. Mathematical biosciences. 2017;288:130–139.

49. Wiebe EM, Stafford AR, Fredenburgh JC, Weitz JI. Mechanism of catalysis of inhibition of factor IXa by antithrombin in the presence of heparin or pentasaccharide. Journal of Biological Chemistry. 2003;278(37):35767–35774.

50. Tsiang M, Paborsky L, Li WX, Jain A, Mao C, Dunn K, et al. Protein engineering thrombin for optimal specificity and potency of anticoagulant activity in vivo. Biochemistry. 1996;35(51):16449–16457.
