# Supplementary material for: A mathematical model to quantify the effects of platelet count, shear rate, and injury size on the initiation of blood coagulation under venous flow conditions
Source: PLoS One. 2020 Jul 29;15(7):e0235392. doi: 10.1371/journal.pone.0235392 (PMC7390270; doi:10.1371/journal.pone.0235392)
Supplement: Attachment — Submitted filename: Response2.pdf [file pone.0235392.s002.pdf]

A mathematical model to quantify the effects of platelet count, shear rate, and injury size on the initiation of blood coagulation under venous flow conditions

A. Bouchnita, K. Terekhov, P. Nony, Y. Vassilevski, V. Volpert

Authors' responses for the questions of the reviewers

We are grateful to the Reviewer # 1 for his or her further attention to our manuscript. We have taken his remarks into consideration:.

Reviewer #1:

The authors have addressed my comments. However, the answer for the second question can not convince the referee. The Fig.5 B is especially unreasonable. There probably be some technical mistakes. This might be a small problem, which may not influence the other results and conclusions, but a wrong figure is obviously unacceptable. I think this paper could not be accepted unless the authors address this problem.

We have re-run the numerical simulations and improved the post-processing to enhance the quality of Fig. 4 and Fig. 5. The figure quality of Fig. 4 and Fig. 5 is improved and asymmetry is no longer observed in Fig. 5 B.

Another minor comment is that the figures are made negligently, such as the unreadable color map in Fig.4 and Fig.5, the legend in Fig.3 a) 200 10^9, the size of the text in all the figures is careless, especially Fig.8 B) ...

Following the suggestion of the Reviewer, we have enhanced the quality of figures 3, 4, 5, 6, 7, and 8. As suggested, we have changed the legends and labels in Fig. 3 and Fig. 7 by replacing $10^9\ L^{-1}$ with $\times 10^9\ L^{-1}$. The color bars in Fig. 4 and Fig. 5 are made more readable. The font size was made uniform in all Figures including Fig. 8.
